# Survival in the Three Common Variants of Primary Progressive Aphasia: A Retrospective Study in a Tertiary Memory Clinic

**DOI:** 10.3390/brainsci11091113

**Published:** 2021-08-24

**Authors:** Maud Tastevin, Monica Lavoie, Justine de la Sablonnière, Julie Carrier-Auclair, Robert Laforce

**Affiliations:** Clinique Interdisciplinaire de Mémoire, Département des Sciences Neurologiques du CHU de Québec, Faculté de Médecine, Université Laval, Québec, QC G1V 0A6, Canada; monica.lavoie.1@ulaval.ca (M.L.); justine.de-la-sablonniere.1@ulaval.ca (J.d.l.S.); julie.carrier-auclair.1@ulaval.ca (J.C.-A.); robert.laforce@fmed.ulaval.ca (R.L.J.)

**Keywords:** primary progressive aphasia, natural history, mortality, survival, memory clinic

## Abstract

Knowledge on the natural history of the three main variants of primary progressive aphasia (PPA) is lacking, particularly regarding mortality. Moreover, advanced stages and end of life issues are rarely discussed with caregivers and families at diagnosis, which can cause more psychological distress. We analyzed data from 83 deceased patients with a diagnosis of PPA. We studied survival in patients with a diagnosis of logopenic variant (lvPPA), semantic variant (svPPA), or non-fluent variant (nfvPPA) and examined causes of death. From medical records, we retrospectively collected data for each patient at several time points spanning five years before the first visit to death. When possible, interviews were performed with proxies of patients to complete missing data. Results showed that survival from symptom onset and diagnosis was significantly longer in svPPA than in lvPPA (*p* = 0.002) and nfvPPA (*p* < 0.001). No relevant confounders were associated with survival. Mean survival from symptom onset was 7.6 years for lvPPA, 7.1 years for nfvPPA, and 12 years for svPPA. The most common causes of death were natural cardio-pulmonary arrest and pneumonia. Aspiration pneumonia represented 23% of deaths in nfvPPA. In conclusion, this pilot study found significant differences in survival between the three variants of PPA with svPPA showing the longest and nfvPPA showing more neurologically-related causes of death.

## 1. Introduction

Primary progressive aphasias (PPAs) are a group of neurodegenerative diseases characterized by a predominant and progressive deterioration of language, with relative preservation of other cognitive functions over at least two years after the onset of the disease [1]. Since 2011, PPAs have been classified into three variants based on their clinical manifestations: the semantic variant (svPPA), the non-fluent/agrammatic variant (nfvPPA), and the logopenic variant (lvPPA) [2].

Demographic and epidemiological data regarding PPAs are lacking and most estimations are based on Frontotemporal Lobar Degeneration (FTLD) studies. Indeed, PPAs represent 20–40% of FTLD cases [3] with an estimated prevalence between 3.6 and 8.1/100,000 inhabitants [4,5,6]. A recent study suggested a prevalence of 3.1/100,000 (95% confidence interval [2.96–3.23]) from a French database including 2035 PPAs patients followed in tertiary centers [7].

While nfvPPA and svPPA are commonly considered as clinical presentations of FTLD with a predominant FTLD-tau pathology for nfvPPA (64% of cases) and FTLD-TDP-43 for svPPA (80% of cases), it is estimated that 86% of lvPPA are associated with Alzheimer’s pathology [8].

Like all neurodegenerative diseases, the impact of PPA on the functional, socio-economic, and quality of life aspects is significant [9,10]. In addition, at diagnostic announcement, advanced stages and end of life issues are rarely discussed with families and caregivers. Moreover, no pertinent guidelines are available for assisting the medical team in prognosis announcement, which can have a significant psychological impact for patients and their families [11,12]. 

Although some authors have studied the natural history of FTLD variants, survival analyses remain scarce in the literature especially for PPAs. In the most recent mixed effects meta-analysis of survival in FTLD published in 2016 by Kansal et al. [13], the mean and median survival in svPPA variant were respectively 7.45 and 12.22 years. For nfvPPA, mean and median survival were 7.69 and 8.11 years, respectively [13]. To date, no study included the three PPA variants in survival analyses and epidemiological and survival data are still unknown for lvPPA. Therefore, it is essential to improve our knowledge of the natural history of PPAs, so that patients and their families can be informed about the onset and management of the different clinical manifestations as well as be properly prepared for end of life issues. The aim to the present pilot study was to analyze and compare survival data between the three PPAs variants and to describe causes of death.

## 2. Materials and Methods

### 2.1. Subjects

We conducted a retrospective study including deceased patients with a diagnosis of PPA that had been followed at our tertiary memory clinic over the past twenty years (*n* = 83). Initial diagnostic evaluation was obtained by a neurologist, a geriatrician or a neuropsychiatrist with a strong clinical experience in cognitive disorders. Diagnosis was based on an extensive clinical and paraclinical evaluation including speech and neuropsychological examination, structural (MRI–dementia protocol) and molecular neuroimaging (FDG-PET), or cerebrospinal fluid AD biomarkers (aB1-42, total-tau, phospho-tau). For all patients recruited after 2011, diagnosis was established according to the clinical criteria by Gorno-Tempini et al. [2]. According to these criteria, svPPA is associated with impaired confrontation naming and single-word comprehension. Additionally, patients may show impaired object knowledge as well as surface dyslexia or dysgraphia. In this variant, repetition and speech production are usually spared. Secondly, patients with nfvPPA must show apraxia of speech and/or agrammatism in speech production. Comprehension of syntactically complex sentences can also be impaired. Spared single-word comprehension and object knowledge is expected. Finally, lvPPA is defined by impaired single-word retrieval in naming and spontaneous speech as well as impaired sentence repetition, particularly for long sentences. Patients may also produce phonologic errors. In this variant, single-word comprehension, object knowledge, grammar and speech production are usually spared. Patients recruited before 2011 were screened and reclassified a posteriori using these diagnostic criteria. Only patients for whom the date of death was not available were excluded from this study, otherwise this study recruited all deceased PPA patients. 

### 2.2. Data Collection

**Figure 1:**
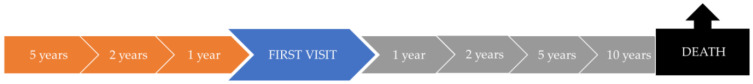
Follow-up strategy for data extraction.

For each patient, data were collected from medical records at several time periods five years before the first visit until death that is at five years, two years and one year before the first visit, the day of the first visit, as well as one year, two years, five years and 10 years after the first visit. Our follow-up strategy is illustrated in Figure 1.

Three of the authors (psychiatrist, neurologist, and a speech therapist) independently screened and extracted the data for all 83 patients. Subsequently, each diagnosis and data were reviewed, and disagreements were resolved by a consensus discussion between the authors.

Data were collected using a standard electronic form to ensure consistency of the appraisals and diagnosis for each patient.

For the present study, the following data were extracted from the database:

1. Socio-demographic data: gender, years of education;
2. Clinical data: diagnosis, MMSE at onset, age at symptoms onset, age at first visit, age at diagnosis, age of death, cause of death, duration from onset to diagnosis, duration from onset to first visit, duration from first visit to diagnosis, disease duration from diagnosis, and disease duration from onset.

When possible, data were validated and corroborated using a semi-structured telephone interview with caregivers. For the present study, 19 interviews were available.

The presence of comorbidities (i.e., cardiovascular risk factors and pulmonary risk factors) as well as intake of medication for cardiovascular conditions (hypertension, diabetes, dyslipidemia) and psychological disorders (anxiety, depression, psychosis, hallucinations, etc.) were extracted and all three groups were similar. Of note, some comorbidity data were not available, and we therefore only focused on those related to cardiovascular and pulmonary data.

### 2.3. Statistical Analyses

Statistical analyses were performed using the R Core Team software [14]. Mean and standard deviations were used to present patient characteristics whereas we used proportions to describe causes of death. A Khi-2 test was conducted for qualitative data. A one-way analysis of variance ANOVA was conducted to compare mean values between the three PPAs variants. A Kaplan–Meier analysis was performed to analyze survival and completed by log rank tests to examine survival curves across diagnostic groups. Cox’s proportional hazard regression were performed to evaluate the effect of possible confounders. All statistical analyses were performed two-sided, and a *p* value of <0.05 was considered as significant.

## 3. Results

### 3.1. Patient Characteristics

Sociodemographic and clinical characteristics of the patients are described in Table 1. Eighty-three patients were included in the analyses: 35 lvPPA, 18 svPPA, and 30 nfvPPA. The proportion of male and female was equivalent in each group and there was no statistically significant difference between the three PPA variants. MMSE scores at first visit and education level were also not significantly different across the variants. However, svPPA patients with more years of education (>12 years) were overrepresented compared to nfvPPA and lvPPA (*p* = 0.012).

Mean age of onset was 69.05 ± 10.8 years for lvPPA and 70.13 ± 6.9 for nfvPPA. The mean age of onset was earlier for svPPA (64.38 ± 7.8 years), but no statistically significant difference was found compared to lvPPA (*p* = 0.17) and nfvPPA (*p* = 0.08). No significant differences were found regarding age of death which occurred after 74 years-old for each subgroup. Mean age at diagnosis did not significantly differ between the three PPA variants.

### 3.2. Disease Duration

**Figure 2:**
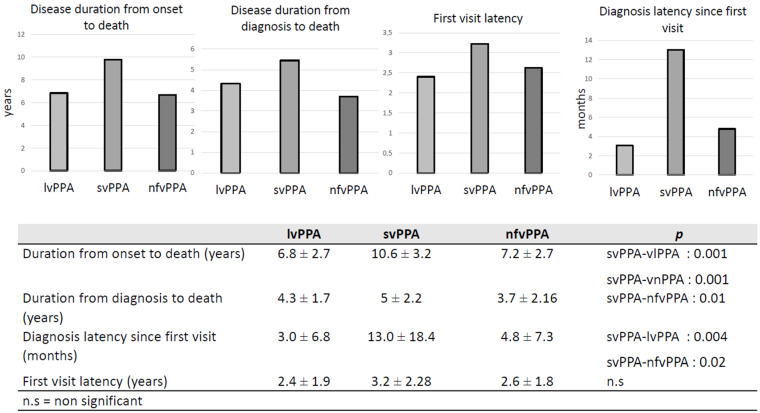
Disease duration.

**Figure 3:**
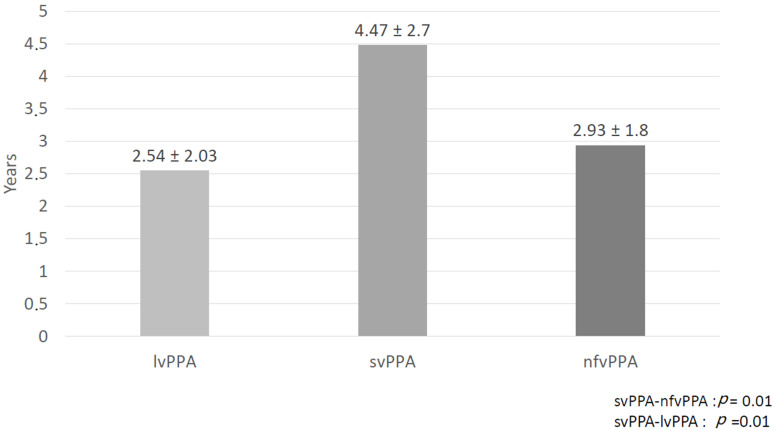
Diagnosis latency since onset.

Several interval times were analyzed to describe and compare disease duration. Duration from onset was significantly longer for svPPA (*p* = 0.001 versus nfvPPA and lvPPA). Disease duration since diagnosis was also longer for svPPA but a statistically significant difference was only observed when compared with nfvPPA (*p* = 0.01). Diagnosis latency, which corresponds to the mean time needed to provide a diagnosis, was significantly longer for svPPA versus other variants. A mean of 13 ± 18 months was necessary to establish a diagnosis of svPPA after the first visit (see Figure 2) and diagnostic latency from first symptoms was 4.47 ± 2.03 years (see Figure 3). No differences were found between the variants in the interval between first symptoms and first medical visit.

### 3.3. Survival Analyses

Mean survival time is summarized in Table 2. Mean and estimated median survival time in patients with svPPA were respectively 12 years and 10 years from onset to death and 7.3 years and 7.5 years from diagnosis to death. Mean survival time in nfvPPA and lvPPA were respectively 7.1 and 7.6 years from onset to death, and 5.6 and 4.7 years from onset to diagnosis. Estimated median survival time was the same for both variants with six years from onset and five years from diagnosis. Causes of death were only available for 34 patients. The most common causes were natural cardio-pulmonary arrest (26.4%), followed by pneumonia (23.52%), cachexia (14.7%), and bedsores infections (11.7%). Major adverse cardiovascular events including stroke, cardiac infraction and systemic embolism represented 11.7% of causes. Half of the pneumonia were of the aspiration subtype and this accounted for 23% of deaths in nfvPPA.

**Figure 4:**
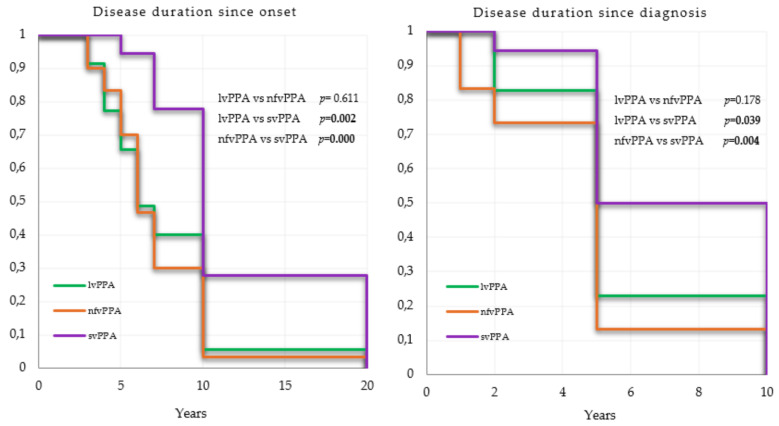
Kaplan–Meier survival curves.

Survival time since onset and diagnosis were significantly longer in svPPA than in lvPPA (*p* = 0.02 and *p* = 0.04, respectively) and nfvPPA (*p* < 0.0001 and *p* = 0.004, respectively) (see Figure 4). A poor association was observed between age of onset and survival from onset in lvPPA with a hazard ratio (HR) next to 1 (HR = 1.008, *p* = 0.019), not pertinent for interpretation (see Table 3). No relevant confounders were associated with survival since diagnosis (see Table 4). 

## 4. Discussion

The aim of this work was to study survival in a group of deceased PPA patients that have been followed at our memory clinic. To date, only five studies on survival in nfvPPA and svPPA were published [15,16,17,18,19]. Among these studies, only three compared the two variants. Moreover, publication date was before the most recent classification criteria by Gorno-Tempini and colleagues. Since 2011, no PPA survival analysis has been done and therefore no study included patients with lvPPA. To our knowledge, this is the first survival study including patients with the three PPA variants.

Despite the small sample size, PPA groups were representative of current practice, where approximately 42% of patients present with lvPPA, 36% with nfvPPA, and 22% with svPPA. Magnin et al. in 2016 [7], compared the demographical data across 2035 PPA subjects. They proposed three samples of patients. Sample 1 included all participants (*n* = 2035) and Sample 2 (*n* = 67) was a subgroup from Sample 1 with CSF biormarkers available. Sample 2 was divided between two subgroups: nfvPPA/ lvPPA/unclassifiable PPA and svPPA. Sample 3 (*n* = 97) was divided between lvPPA, svPPA, and nfvPPA, and the CSF biomarkers were available for all of them. In Sample 1, the proportion of svPPA patients represented 28.1% of the entire sample. Moreover, the repartition was comparable across each sample with less svPPA patients [7]. Our sample also showed a smaller proportion of svPPA patients compared to the two other variants. Moreover, the authors estimated that nfvPPA/lvPPA/unclassifiable PPA were more frequent than svPPA (2.2 versus 0.8/100,000 inhabitants; *p* < 0.00001) [7].

In this study, patients with svPPA were younger than lvPPA and nfvPPA patients with a mean age at onset of 64 years-old, which is similar to that found in the three survival studies cited earlier. However, no statistically significant differences across the PPA groups were found, which was also the case in the studies by Hodges et al. (2003), Nunnemann et al. (2011), and Roberson et al. (2005) [15,17,18]. On the other hand, Kertesz et al. (2007) showed a statistically significant younger age of onset in svPPA patients compared to nfvPPA patients [16]. The same profile was highlighted concerning age at diagnosis. Age at diagnosis was also younger in the eight natural studies of PPA including the three variants (between 60 and 70 years) and no differences were observed across groups [19,20,21,22,23,24,25,26]. Absence of differences could be explained by small sample sizes. Indeed, no data were available for sample sizes over 150 subjects. In Sample 1 (*n* = 2035) of the multicentric epidemiologic study published by Magnin et al. (2016) [7], the nfvPPA/lvPPA/unlassifiable PPA group was significantly older at disease onset and at diagnosis than the svPPA group (*p* < 0.00001). There was no significant differences in level of education or gender. Compared to typical AD, in svPPA patients, male predominance occurred after the age of 80, the level of education was higher, and the age of onset was younger (71.6 versus 78.61 years old), all differences being statistically significant with a *p* < 0.0001. In Sample 3 (*n* = 97), svPPA patients were also younger at disease onset than lvPPA patients (59.48 years-old versus 63.72 years-old) and no differences were observed on gender and age of education. After CSF biomarkers stratification, no significant difference was observed for age at onset, gender, or level of education between the “PPA-AD” group and the “PPA–not “AD” group [7].

In this study, no significant differences were observed across MMSE scores. In a study by Ulugut et al. published in 2021 [21], MMSE score also did not differ significantly among the PPA variants. However, lvPPA patients performed worse on executive and visuospatial specific testing and svPPA performed worse on the verbal memory test at baseline. These findings must, however, be interpreted with caution as cognitive assessment can be strongly impacted by language abilities especially on global scores (MoCA and MMSE). In clinical practice, it is frequent to observe a clinical dissociation between performance on cognitive tests and level of functioning on collateral history. None of the PPA patients were tested using specific assessment and neuropsychological tools that take into account language impairments [27].

In the present study, diagnostic latencies since onset and since the first visit were significantly longer for svPPA versus the other variants. Especially, the diagnostic latency since onset was 4.47 years in svPPA versus 2.54 years in lvPPA and 2.93 years in nfvPPA. Although latencies were not explored in PPA survival studies, several other observational studies have found similar results. Indeed, in the analysis of Sample 3, Magnin et al. (2016) found that the delay between first symptoms and PPA diagnosis was longer in svPPA patients (4.48 years) compared to lvPPA (3.02 years) and nfvPPA (2.26 years) [7]. In the study published by Van Langenhove et al. (2016), symptoms duration at baseline was 4.4 years for svPPA, compared to 2.3 and 3.5 years in nfvPPA and lvPPA, respectively [22]. Hseish et al. (2012) also found that svPPA had a mean disease duration of 4.2 years at time of diagnosis, compared with 2.3 and 3.9 years for nfvPPA and lvPPA [26]. These results could be explained by a more challenging recognition of the disease by the family and the physicians. Indeed, loss of semantic knowledge can be masked by a more fluent profile and patients are, therefore, more able to develop compensation mechanisms at the onset of disease. Moreover, it can also be masked by the first symptoms being characterized as a predominant memory and behavioral presentation [21]. 

Our survival results were compatible with findings from a meta-analysis published by Kansal et al. in 2016, with a median survival estimated at 10 years for svPPA (Kansal et al. showed a median svPPA survival of 12 years). In this study, we also found a significantly longer survival time in svPPA patients. However, Kansal et al. results revealed a significant difference only in median survival, whereas mean survival between svPPA and nfvPPA patients did not reach statistical significance [13]. The three survival studies comparing svPPA and nfvPPA did not show differences on survival time [15,16,17]. Moreover, contrary to Kansal et al., we found higher mean survival times than median survival times for svPPA and nfvPPA. The authors underlined this asymmetry between median and mean survival findings in svPPA results. In their discussion, Kansal et al. suggested an artefact in the analysis due to the heterogeneity the included studies (sampling methods and regional context) but they also proposed an interesting hypothesis where presence of a negative or positive skew could be a statistical reflection of the survival profile. A negative skew could concern a disease with young- or mid-life onset, a sufficiently long course, with few premature deaths. In contrast, a positive skew was more likely when a disease was characterized by a very short course, as the outliers are those with unusually long survival. It is important to consider that the meta-analysis was first limited by the small number of studies included, with only three comparing nfvPPA and svPPA. In the study by Nunnemann et al. [17], median survival in the svPPA group could not be defined because less than half of the patients had died at the end of the observational period. Moreover, in the three studies, the sample size of died patients was extremely limited with only nine svPPA and eight nfvPPA for Hodges et al. (2003) [15], three svPPA and seven nfvPPA for Nunnemann et al. (2011) [17], twelve svPPA and seven nfvPPA for Roberson et al. (2005) [18].

According to the WHO definition, years of life lost (YLL) is the age at which deaths occur by giving greater weight to deaths at younger age and lower weight to deaths at older age. The years of life lost (percentage of total) indicator measures the YLL due to a cause as a proportion of the total YLL lost in the population due to premature mortality. We did not include YLL in our survival analyses. It could have been interesting especially for patients and their family to know which variant is associated with premature mortality. In the meta-analysis of Kansal et al. (2016), mean YLL was significantly higher in svPPA compared to nfvPPA (13.56 years versus 10.54 years). These results could also be overestimated for svPPA because only premature deaths were analyzed for svPPA in the study of Nunnemann et al. (2011), given that less than half of the patients had died at the end of the observational period.

Finally, causes of death in PPA are poorly described in the current literature. In this study, we were only able to obtain causes for 34 patients, mostly by interviews with the caregivers. Our results were similar to the ones of Nunnemann et al. (2011) [17] with a predominance of respiratory system disorder including aspiration pneumonia, circulatory system disorder and cachexia. It has been our experience that a small proportion of PPA patients develop Progressive Supranuclear Palsy and/or Corticobasal Syndrome. These patients tend to show a poorer evolution over time. However, this study did not specifically address this or intend to compare AD with PSP and/ or CBS.

The main limitation of this pilot work was undoubtedly the small sample size, however, of higher or comparable size to that of studies already published. Our study was also the first to include lvPPA patients. Future multicentric studies should be conducted on the three PPA variants to obtain results for larger sample sizes. Moreover, in this study, few data were available regarding causes of death. Indeed, most patients died when in long term care homes and therefore were not admitted in the hospital at the time of death. Moreover, many came from all across the province of Quebec and their full medical record was not available to us. Finally, most diagnoses were not confirmed by pathological autopsy and misdiagnosis cannot be excluded for patients diagnosed prior to the 2011 criteria. However, our data extraction strategy was independently realized by a pluridisciplinary team experienced in the classification of PPAs, and secondarily completed by a consensus discussion to control for management of clinical misdiagnosis.

## 5. Conclusions

This is the first study to analyze survival data across the three variants of PPA according to Gorno-Tempini’s criteria [2]. Our preliminary results tend to highlight a specific profile for svPPA patients in comparison to the two other variants. Patients suffering from svPPA seem to get a confirmed diagnosis later than the others but svPPA remains characterized by the best survival time. More survival analyses, integrating the most recent diagnostic criteria, all phenotypes and multicentric databases will be necessary to confirm our findings. Moreover, more complete survival analyses including median and mean survival time, YLL, comorbidities and treatments could offer better prevention information for patients and their caregivers by taking account premature death and their causes. To date, these preliminary findings already provide precious data to better prepare them to the progression of the disease and end stages of life.

## Figures and Tables

**Table 1:** Patient characteristics.

	lvPPA (*n* = 35)	nfvPPA (*n* = 30)	svPPA (*n* = 18)	*p*
Male/Female	19/16	15/15	9/9	n.s
Education years (mean ± SD)	11.7 ±4.4	11.03 ± 4.2	13.17 ± 4.1	n.s
Education level (>12 years)	15	9	12	0.026
MMSE 1st visit (mean ± SD)	21.03 ± 5.9	21.67 ± −6.8	22.35 ± 7.4	n.s
Age of onset (mean ± SD)	69.05 ± 10.8	70.13 ± 6.9	64.38 ± 7.8	n.s
Age at diagnosis (mean ± SD)	71.65 ± 10.22	73.1 ± 6.79	68.68 ± 8.5	n.s
Age of death (mean ± SD)	75.90 ± 9.5	76.8 ± 6.3	74.11 ± 8.3	n.s

n.s = not significant. SD = standard deviation.

**Table 2:** Mean and estimated median survival time (years).

	Mean Survival		Estimated Median Survival	
	Since Onset	Since Diagnosis	Since Onset	Since Diagnosis
lvPPA (*n* = 35)	7.6 CI95%: 6.2–8.9	5.6 CI95%: 4.5–6.5	6 CI95%: 5.0–10.0	5 CI95%: 0.0–10
nfvPPA (*n* = 30)	7.1 CI95%: 5.9–8.3	4.7 CI95%: 3.7–7.6	6 CI95%: 6.00–7.00	5 CI95%: 0.0–10.0
svPPA (*n* = 18)	12 CI95%: 9.5–14.4	7.3 CI95%: 6.0–8.6	10 CI95%: 0.0–20.0)	7.5 CI95%: 5.0–10.0

CI = Confidence interval.

**Table 3:** Hazard ratios since onset.

	lvPPA		nfvPPA		svPPA	
	HR (95%CI)	*p*-Value	HR (95%CI)	*p*-Value	HR (95%CI)	*p*-Value
MMSE 1st visit	0.96 (0.89–1.03)	0.30	0.96 (0.89–1.02)	0.21	0.99 (0.89–1.10)	0.90
Age of onset	1.05 (1.00–1.08)	0.02	1.03 (0.95–1.11)	0.52	0.96 (0.86–1.07)	0.43
Years of education	1.02 (0.94–1.11)	0.56	1.02 (0.88–1.17)	0.84	0.98 (0.82–1.16)	0.81
Gender	1.36 (0.56–3.32)	0.50	0.82 (0.33–2.08)	0.68	0.46 (0.13–1.64)	0.2

HR = Hazard ratio; CI = Confidence interval.

**Table 4:** Hazard ratios since diagnosis.

	lvPPA		nfvPPA		svPPA	
	HR (95%CI)	*p*-Value	HR (95%CI)	*p*-Value	HR (95%CI)	*p*-Value
MMSE 1st visit	0.98 (0.92–1.06)	0.66	0.98 (0.87–1.10)	0.39	0.99 (0.89–1.10)	0.91
Age of onset	1.01 (0.98–1.06)	0.48	1.02 (0.95–1.11)	0.52	1.00 (0.91–1.09)	0.99
Years of education	1.02 (0.94–1.10)	0.57	0.98 (0.87–1.10)	0.74	0.99 (0.82–1.20)	0.98
Gender	0.80 (0.36–1.81)	0.60	1.19 (0.49–2.84)	0.70	1.12 (0.28–4.52)	0.87

HR = Hazard ratio; CI = Confidence interval.

## Data Availability

The data that support the findings of this study are available from the corresponding author upon reasonable request.
